# The genome sequence of the Light Brocade, *Lacanobia w-latinum *(Hufnagel, 1766)

**DOI:** 10.12688/wellcomeopenres.19653.1

**Published:** 2023-07-11

**Authors:** Douglas Boyes, Peter W.H. Holland, University of Oxford and Wytham Woods Genome Acquisition Lab, Darwin Tree of Life Barcoding collective, Wellcome Sanger Institute Tree of Life programme, Wellcome Sanger Institute Scientific Operations: DNA Pipelines collective, Tree of Life Core Informatics collective, Darwin Tree of Life Consortium

**Affiliations:** 1 UK Centre for Ecology & Hydrology, Wallingford, England, UK; 2 University of Oxford, Oxford, England, UK

**Keywords:** Lacanobia w-latinum, Light Brocade, genome sequence, chromosomal, Lepidoptera

## Abstract

We present a genome assembly from an individual male *Lacanobia w-latinum *(the Light Brocade; Arthropoda; Insecta; Lepidoptera; Noctuidae). The genome sequence is 903.9 megabases in span. Most of the assembly is scaffolded into 31 chromosomal, including the Z sex chromosome. The mitochondrial genome has also been assembled and is 15.38 kilobases in length. Gene annotation of this assembly on Ensembl identified 21,592 protein coding genes.

## Species taxonomy

Eukaryota; Metazoa; Eumetazoa; Bilateria; Protostomia; Ecdysozoa; Panarthropoda; Arthropoda; Mandibulata; Pancrustacea; Hexapoda; Insecta; Dicondylia; Pterygota; Neoptera; Endopterygota; Amphiesmenoptera; Lepidoptera; Glossata; Neolepidoptera; Heteroneura; Ditrysia; Obtectomera; Noctuoidea; Noctuidae; Hadeninae; *Lacanobia; Lacanobia w-latinum* (Hufnagel, 1766) (NCBI:txid987426).

## Background

Climate change can have a range of impacts on insect populations including effects on distribution, dispersal, abundance, and life cycle parameters. These may be induced directly, through insects responding to changes in temperature or precipitation, or indirectly through responses to changes in plant communities, competitors, predators, parasites or pathogens ( Cohen *et al.*, 2018; McCarty *et al.*, 2017). A progressive shift of flight season to earlier in the year has been noted for many, though not all, species of Lepidoptera in Britain and Ireland, in parallel to climate change ( Randle *et al.*, 2019). The Light Brocade, *Lacanobia w-latinum*, is a species for which the flight period has clearly advanced in southern Britain over the past 50 years, with records from 1970–1⁠979 peaking in mid-June compared to late May for 2000–⁠2016 ( Randle *et al.*, 2019). 

*Lacanobia w-latinum* is a striking member of the family Noctuidae. The forewings of the adult moth are neatly patterned with russet-brown and white markings, including a broad silvery cross-band following the termen of the wing and pronounced ‘dog-tooth’ terminal markings typical of this genus. The moth is distributed widely across Europe with many records from the UK, Austria, Switzerland, Netherlands, France, Denmark and southern regions of Sweden. There are also scattered records further east from Ukraine, Russia, Iran, Kazakhstan and Uzbekistan ( GBIF Secretariat, 2022). In Britain, the moth is found predominantly south of a line running from the Bristol Channel to the Wash in calcareous grasslands, downland and heathland habitats, although it can also be found in open woodland and gardens ( Randle *et al.*, 2019). It has not been recorded from Ireland. The larvae are nocturnal and feed in late summer on the leaves of common broom ( *Cytisus scoparius*), dyer’s greenweed ( *Genista tinctoria*), knotgrass ( *Polygonum aviculare*) and some other low growing plants; the pupal stage overwinters ( Bretherton *et al.*, 1983).

A complete genome sequence of *Lacanobia w-latinum* may facilitate studies into adaptations to calcareous habitats and genetic responses to climate change, and will add to the growing set of genomic resources for studying lepidopteran evolution.

## Genome sequence report

**Figure 1.**
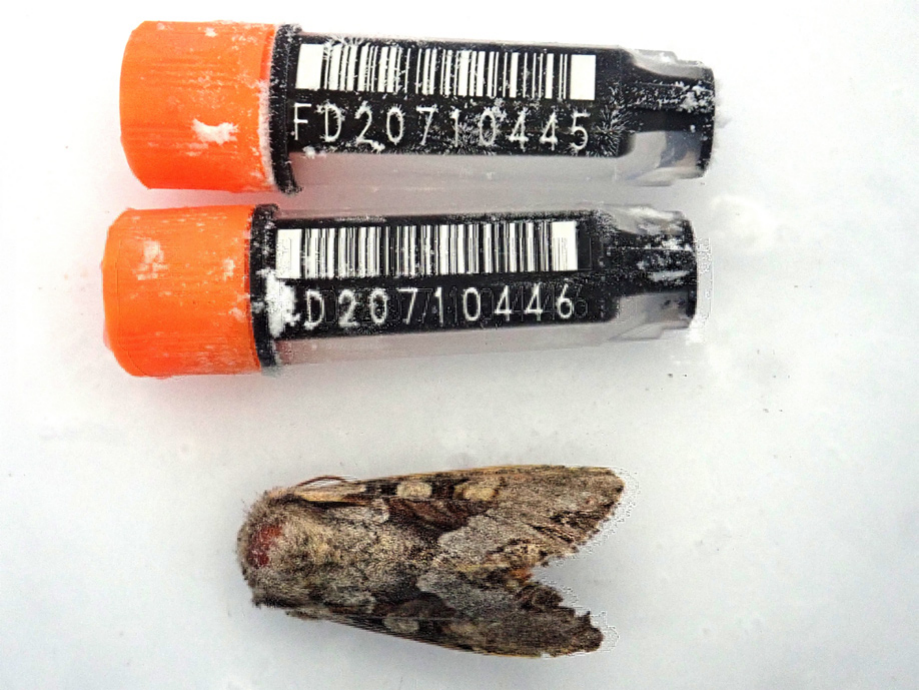
Photograph of the *Lacanobia w-latinum* (ilLacWlai2) specimen used for genome sequencing.

The genome was sequenced from one male *Lacanobia w-latinum* ( Figure 1) collected from Wytham Woods, Oxfordshire (51.77, –1.34). A total of 48-fold coverage in Pacific Biosciences single-molecule HiFi long reads was generated. Primary assembly contigs were scaffolded with chromosome conformation Hi-C data. Manual assembly curation corrected 105 missing joins or mis-joins and removed 21 haplotypic duplications, reducing the assembly length by 0.47% and the scaffold number by 83.35%, and increasing the scaffold N50 by 6.94%.

**Figure 2.**
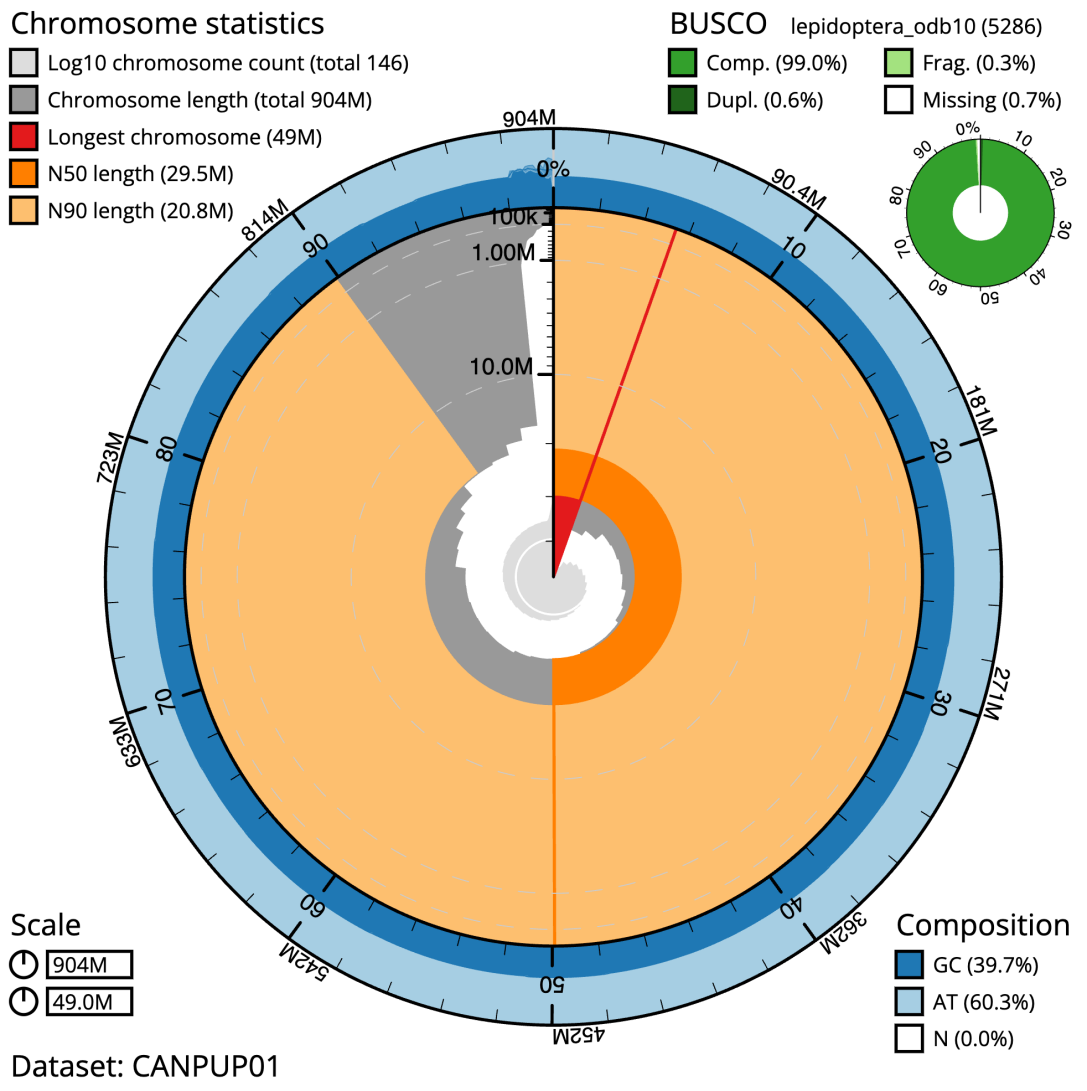
Genome assembly of *Lacanobia w-latinum*, ilLacWlai2.1: metrics. The BlobToolKit Snailplot shows N50 metrics and BUSCO gene completeness. The main plot is divided into 1,000 size-ordered bins around the circumference with each bin representing 0.1% of the 903,922,692 bp assembly. The distribution of scaffold lengths is shown in dark grey with the plot radius scaled to the longest scaffold present in the assembly (48,999,040 bp, shown in red). Orange and pale-orange arcs show the N50 and N90 scaffold lengths (29,461,643 and 20,845,940 bp), respectively. The pale grey spiral shows the cumulative scaffold count on a log scale with white scale lines showing successive orders of magnitude. The blue and pale-blue area around the outside of the plot shows the distribution of GC, AT and N percentages in the same bins as the inner plot. A summary of complete, fragmented, duplicated and missing BUSCO genes in the lepidoptera_odb10 set is shown in the top right. An interactive version of this figure is available at https://blobtoolkit.genomehubs.org/view/Lacanobia wlatinum/dataset/CANPUP01/snail.

The final assembly has a total length of 903.9 Mb in 145 sequence scaffolds with a scaffold N50 of 29.5 Mb ( Table 1). Most (99 %) of the assembly sequence was assigned to 31 chromosomal-level scaffolds, representing 30 autosomes and the Z sex chromosome. Chromosome-scale scaffolds confirmed by the Hi-C data are named in order of size ( Figure 2– Figure 5; Table 2). While not fully phased, the assembly deposited is of one haplotype. Contigs corresponding to the second haplotype have also been deposited. The mitochondrial genome was also assembled and can be found as a contig within the multifasta file of the genome submission.

**Table 1.** Genome data for *Lacanobia w-latinum*, ilLacWlai2.1.

Project accession data		
Assembly identifier	ilLacWlai2.1	
Species	*Lacanobia w-latinum*	
Specimen	ilLacWlai2	
NCBI taxonomy ID	987426	
BioProject	PRJEB56249	
BioSample ID	SAMEA10979194	
Isolate information	ilLacWlai2, male: whole organism (DNA sequencing, Hi-C scaffolding, RNA sequencing)	
Assembly metrics *		***Benchmark***
Consensus quality (QV)	64.7	*≥ 50*
*k*-mer completeness	100%	*≥ 95%*
BUSCO **	C:99.0%[S:98.4%,D:0.6%], F:0.3%,M:0.7%,n:5,286	*C ≥ 95%*
Percentage of assembly mapped to chromosomes	99%	*≥ 95%*
Sex chromosomes	Z chromosome	*localised homologous pairs*
Organelles	Mitochondrial genome assembled	*complete single alleles*
Raw data accessions		
PacificBiosciences SEQUEL II	ERR10355967, ERR10355968	
Hi-C Illumina	ERR10297863	
PolyA RNA-Seq Illumina	ERR10908607	
Genome assembly		
Assembly accession	GCA_947578705.1	
*Accession of alternate haplotype*	GCA_947579085.1	
Span (Mb)	903.9	
Number of contigs	272	
Contig N50 length (Mb)	22.0	
Number of scaffolds	145	
Scaffold N50 length (Mb)	29.5	
Longest scaffold (Mb)	49	
Genome annotation		
Number of protein-coding genes	21,592	
Number of gene transcripts	21,806	

* Assembly metric benchmarks are adapted from column VGP-2020 of “Table 1: Proposed standards and metrics for defining genome assembly quality” from ( Rhie *et al.*, 2021). ** BUSCO scores based on the lepidoptera_odb10 BUSCO set using v5.3.2. C = complete [S = single copy, D = duplicated], F = fragmented, M = missing, n = number of orthologues in comparison. A full set of BUSCO scores is available at https://blobtoolkit.genomehubs.org/view/ilLacWlai2.1/dataset/CANPUP01/busco.

**Figure 3.**
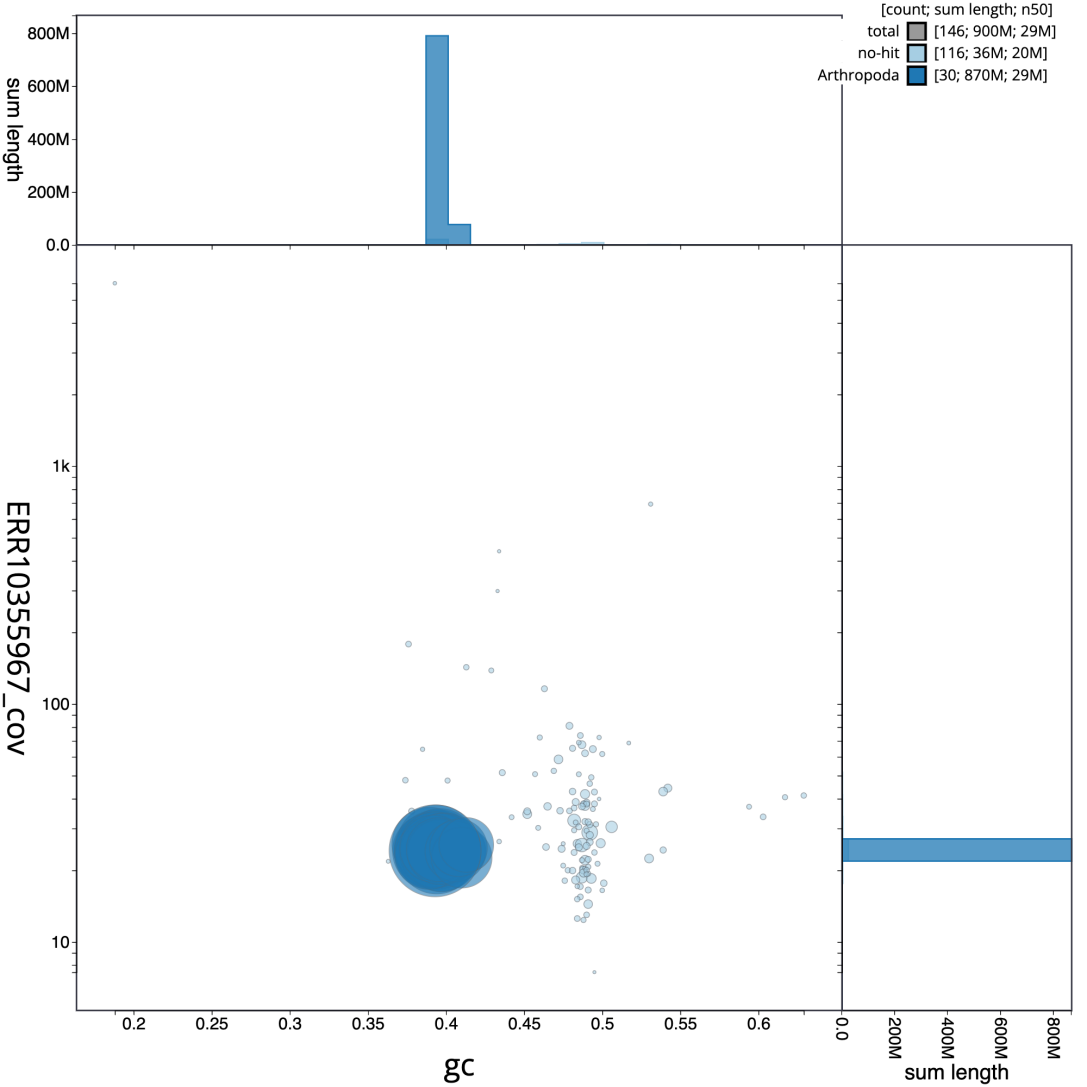
Genome assembly of *Lacanobia w-latinum*, ilLacWlai2.1: BlobToolKit GC-coverage plot. Scaffolds are coloured by phylum. Circles are sized in proportion to scaffold length. Histograms show the distribution of scaffold length sum along each axis. An interactive version of this figure is available at https://blobtoolkit.genomehubs.org/view/Lacanobia wlatinum/dataset/CANPUP01/blob.

**Figure 4.**
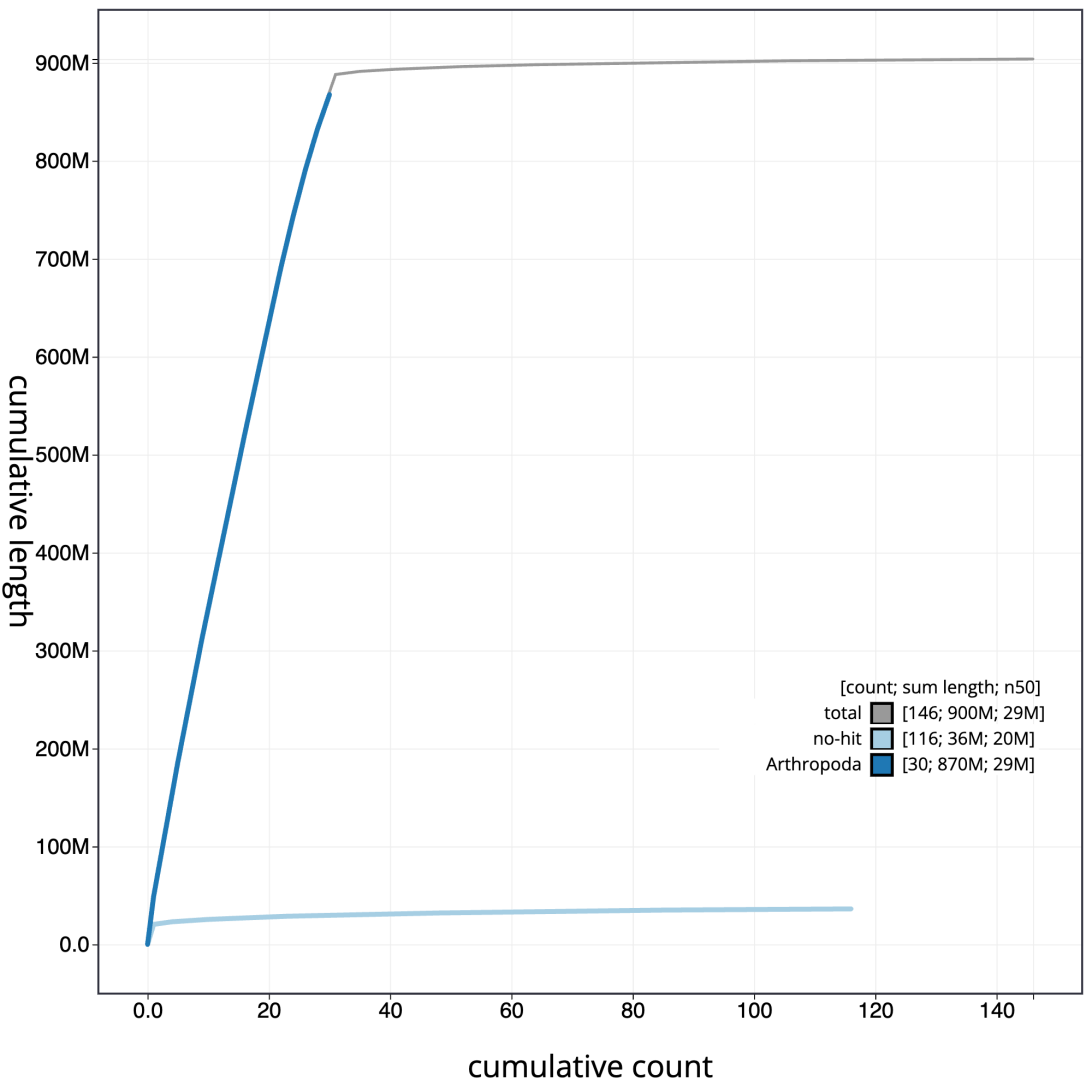
Genome assembly of *Lacanobia w-latinum*, ilLacWlai2.1: BlobToolKit cumulative sequence plot. The grey line shows cumulative length for all scaffolds. Coloured lines show cumulative lengths of scaffolds assigned to each phylum using the buscogenes taxrule. An interactive version of this figure is available at https://blobtoolkit.genomehubs.org/view/Lacanobia wlatinum/dataset/CANPUP01/cumulative.

**Figure 5.**
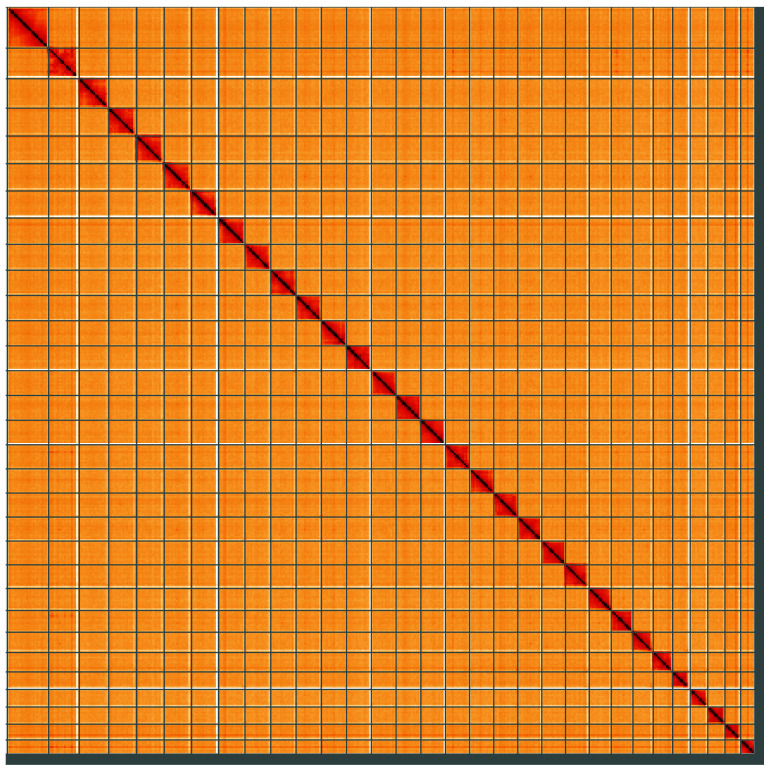
Genome assembly of *Lacanobia w-latinum*, ilLacWlai2.1: Hi-C contact map of the ilLacWlai2.1 assembly, visualised using HiGlass. Chromosomes are shown in order of size from left to right and top to bottom. An interactive version of this figure may be viewed at https://genome-note-higlass.tol.sanger.ac.uk/l/?d=GCV-ScdiSVyHt69lEDLeMQ.

**Table 2.** Chromosomal pseudomolecules in the genome assembly of *Lacanobia w-latinum*, ilLacWlai2.

INSDC accession	Chromosome	Length (Mb)	GC%
OX388023.1	1	36.39	39.5
OX388024.1	2	34.99	39.5
OX388025.1	3	33.07	39.5
OX388026.1	4	32.83	39.0
OX388027.1	5	32.52	39.5
OX388028.1	6	32.18	39.5
OX388029.1	7	31.21	39.0
OX388030.1	8	30.75	39.5
OX388031.1	9	29.18	39.0
OX388032.1	10	30.03	39.0
OX388033.1	11	29.98	39.5
OX388034.1	12	29.89	39.5
OX388035.1	13	29.54	39.5
OX388036.1	14	29.46	40.0
OX388037.1	15	29.4	39.0
OX388038.1	16	28.73	39.5
OX388039.1	17	28.7	39.5
OX388040.1	18	28.56	39.5
OX388041.1	19	28.47	40.0
OX388042.1	20	28.26	39.5
OX388043.1	21	28.21	39.5
OX388044.1	22	26.12	39.0
OX388045.1	23	25.74	39.0
OX388046.1	24	24.18	39.5
OX388047.1	25	23.04	39.5
OX388048.1	26	20.87	40.5
OX388049.1	27	20.85	41.0
OX388050.1	28	20.46	39.5
OX388051.1	29	18.72	41.0
OX388052.1	30	16.8	41.5
OX388022.1	Z	49.0	39.5
OX388053.1	MT	0.02	19.0

The estimated Quality Value (QV) of the final assembly is 64.7 with *k*-mer completeness of 100%, and the assembly has a BUSCO v5.3.2 completeness of 99.0% (single = 98.4%, duplicated =0.6%), using the lepidoptera_odb10 reference set ( *n* = 5,286).

Metadata for specimens, spectral estimates, sequencing runs, contaminants and pre-curation assembly statistics can be found at https://links.tol.sanger.ac.uk/species/987426.

## Genome annotation report

The *Lacanobia w-latinum* genome assembly (GCA_947578705.1) was annotated using the Ensembl rapid annotation pipeline ( Table 1; https://rapid.ensembl.org/Lacanobia_wlatinum_GCA_947578705.1/Info/Index). The resulting annotation includes 21,806 transcribed mRNAs from 21,592 protein-coding genes.

## Methods

### Sample acquisition and nucleic acid extraction

The specimen used for genome sequencing was a male *Lacanobia w-latinum* (specimen ID Ox001930, ilLacWlai2), collected using a light trap in Wytham Woods, Oxfordshire (biological vice-county Berkshire), UK (latitude 51.77, longitude –1.34) on 2021-06-16. Douglas Boyes (University of Oxford) collected and identified the specimen. The specimen was snap-frozen on dry ice.

DNA was extracted at the Tree of Life laboratory, Wellcome Sanger Institute (WSI). The ilLacWlai2 sample was weighed and dissected on dry ice with tissue set aside for Hi-C sequencing. Tissue from the whole organism was cryogenically disrupted to a fine powder using a Covaris cryoPREP Automated Dry Pulveriser, receiving multiple impacts. High molecular weight (HMW) DNA was extracted using the Qiagen MagAttract HMW DNA extraction kit. HMW DNA was sheared into an average fragment size of 12–20 kb in a Megaruptor 3 system with speed setting 30. Sheared DNA was purified by solid-phase reversible immobilisation using AMPure PB beads with a 1.8X ratio of beads to sample to remove the shorter fragments and concentrate the DNA sample. The concentration of the sheared and purified DNA was assessed using a Nanodrop spectrophotometer and Qubit Fluorometer and Qubit dsDNA High Sensitivity Assay kit. Fragment size distribution was evaluated by running the sample on the FemtoPulse system.

RNA was extracted from tissue of ilLacWlai2 in the Tree of Life Laboratory at the WSI using TRIzol, according to the manufacturer’s instructions. RNA was then eluted in 50 μl RNAse-free water and its concentration assessed using a Nanodrop spectrophotometer and Qubit Fluorometer using the Qubit RNA Broad-Range (BR) Assay kit. Analysis of the integrity of the RNA was done using Agilent RNA 6000 Pico Kit and Eukaryotic Total RNA assay.

### Sequencing

Pacific Biosciences HiFi circular consensus DNA sequencing libraries were constructed according to the manufacturers’ instructions. Poly(A) RNA-Seq libraries were constructed using the NEB Ultra II RNA Library Prep kit. DNA and RNA sequencing was performed by the Scientific Operations core at the WSI on Pacific Biosciences SEQUEL II (HiFi) and Illumina NovaSeq 6000 (RNA-Seq) instruments. Hi-C data were also generated from tissue of ilLacWlai2 that has been set aside, using the Arima2 kit and sequenced on the Illumina NovaSeq 6000 instrument.

### Genome assembly, curation and evaluation

Assembly was carried out with Hifiasm ( Cheng *et al.*, 2021) and haplotypic duplication was identified and removed with purge_dups ( Guan *et al.*, 2020). The assembly was then scaffolded with Hi-C data ( Rao *et al.*, 2014) using YaHS ( Zhou *et al.*, 2023). The assembly was checked for contamination and corrected as described previously ( Howe *et al.*, 2021). Manual curation was performed using HiGlass ( Kerpedjiev *et al.*, 2018) and Pretext ( Harry, 2022). The mitochondrial genome was assembled using MitoHiFi ( Uliano-Silva *et al.*, 2022), which runs MitoFinder ( Allio *et al.*, 2020) or MITOS ( Bernt *et al.*, 2013) and uses these annotations to select the final mitochondrial contig and to ensure the general quality of the sequence.

A Hi-C map for the final assembly was produced using bwa-mem2 ( Vasimuddin *et al.*, 2019) in the Cooler file format ( Abdennur & Mirny, 2020). To assess the assembly metrics, the *k*-mer completeness and QV consensus quality values were calculated in Merqury ( Rhie *et al.*, 2020). This work was done using Nextflow ( Di Tommaso *et al.*, 2017) DSL2 pipelines “sanger-tol/readmapping” ( Surana *et al.*, 2023a) and “sanger-tol/genomenote” ( Surana *et al.*, 2023b). The genome was analysed within the BlobToolKit environment ( Challis *et al.*, 2020) and BUSCO scores ( Manni *et al.*, 2021; Simão *et al.*, 2015) were calculated.

Table 3 contains a list of relevant software tool versions and sources.

**Table 3.** Software tools: versions and sources.

Software tool	Version	Source
BlobToolKit	4.1.5	https://github.com/blobtoolkit/blobtoolkit
BUSCO	5.3.2	https://gitlab.com/ezlab/busco
Hifiasm	0.16.1-r375	https://github.com/chhylp123/hifiasm
HiGlass	1.11.6	https://github.com/higlass/higlass
Merqury	MerquryFK	https://github.com/thegenemyers/MERQURY.FK
MitoHiFi	2	https://github.com/marcelauliano/MitoHiFi
PretextView	0.2	https://github.com/wtsi-hpag/PretextView
purge_dups	1.2.3	https://github.com/dfguan/purge_dups
sanger-tol/genomenote	v1.0	https://github.com/sanger-tol/genomenote
sanger-tol/readmapping	1.1.0	https://github.com/sanger-tol/readmapping/tree/1.1.0
YaHS	yahs-1.1.91eebc2	https://github.com/c-zhou/yahs

### Genome annotation

The BRAKER2 pipeline ( Brůna *et al.*, 2021) was used in the default protein mode to generate annotation for the *Lacanobia w-latinum* assembly (GCA_947578705.1). in Ensembl Rapid Release.

### Wellcome Sanger Institute – Legal and Governance

The materials that have contributed to this genome note have been supplied by a Darwin Tree of Life Partner. The submission of materials by a Darwin Tree of Life Partner is subject to the **‘Darwin Tree of Life Project Sampling Code of Practice’**, which can be found in full on the Darwin Tree of Life website here. By agreeing with and signing up to the Sampling Code of Practice, the Darwin Tree of Life Partner agrees they will meet the legal and ethical requirements and standards set out within this document in respect of all samples acquired for, and supplied to, the Darwin Tree of Life Project.

Further, the Wellcome Sanger Institute employs a process whereby due diligence is carried out proportionate to the nature of the materials themselves, and the circumstances under which they have been/are to be collected and provided for use. The purpose of this is to address and mitigate any potential legal and/or ethical implications of receipt and use of the materials as part of the research project, and to ensure that in doing so we align with best practice wherever possible. The overarching areas of consideration are:

•   Ethical review of provenance and sourcing of the material

•   Legality of collection, transfer and use (national and international) 

Each transfer of samples is further undertaken according to a Research Collaboration Agreement or Material Transfer Agreement entered into by the Darwin Tree of Life Partner, Genome Research Limited (operating as the Wellcome Sanger Institute), and in some circumstances other Darwin Tree of Life collaborators.

## Data Availability

European Nucleotide Archive: *Lacanobia w-latinum* (light brocade). Accession number PRJEB56249; https://identifiers.org/ena.embl/PRJEB56249. ( Wellcome Sanger Institute, 2022) The genome sequence is released openly for reuse. The *Lacanobia w-latinum* genome sequencing initiative is part of the Darwin Tree of Life (DToL) project. All raw sequence data and the assembly have been deposited in INSDC databases. Raw data and assembly accession identifiers are reported in Table 1.

## Author information

Members of the University of Oxford and Wytham Woods Genome Acquisition Lab are listed here: https://doi.org/10.5281/zenodo.4789928.

Members of the Darwin Tree of Life Barcoding collective are listed here: https://doi.org/10.5281/zenodo.4893703.

Members of the Wellcome Sanger Institute Tree of Life programme are listed here: https://doi.org/10.5281/zenodo.4783585.

Members of Wellcome Sanger Institute Scientific Operations: DNA Pipelines collective are listed here: https://doi.org/10.5281/zenodo.4790455.

Members of the Tree of Life Core Informatics collective are listed here: https://doi.org/10.5281/zenodo.5013541.

Members of the Darwin Tree of Life Consortium are listed here: https://doi.org/10.5281/zenodo.4783558.
