# Supplementary material for: The genome sequence of the Light Brocade, Lacanobia w-latinum (Hufnagel, 1766)
Source: Wellcome Open Res. 2023 Jul 11;8:299. [Version 1] doi: 10.12688/wellcomeopenres.19653.1 (PMC10472056; doi:10.12688/wellcomeopenres.19653.1)
Supplement: Peer review history

# Peer review: Reviewer response for version 1

**DOI:** 10.21956/wellcomeopenres.21770.r66255

**Published:** 2023-08-31

**Authors:** Manuela Lopez Villavicencio

**Affiliations:** 1 Institut de Systématique, Evolution, Biodiversité, Muséum National d'Histoire Naturelle, Paris, France

This article reports the genome assembly of a male individual of the European moth *Lacanobia w-latinum*. The assembly included the autosomes, the Z sexual chromosome and the mitochondrial genome. I found really interesting the authors mention that some features of the species, as the flight period, seem to be changing due to climate change.

 The article is clearly written and pleasant to read. It shows convincing evidences for a high-quality assembly based on BUSCO scores. The methods for genome assembly, quality test and Hi-C scaffolding are relevant and up-to-date. There are sufficient details of methods and materials provided to allow replication by others.

 The assembly sequence was assigned to 31 chromosomal-level scaffolds, representing 30 autosomes plus the sexual Z chromosome.

 As I am interested in butterfly genomes, I would have loved to have other interesting information as genome-wide level of heterozygosity (estimated with jellyfish and genomescope). I also think it is important to provide BUSCO quality assessment for the annotation because I usually find over-estimation of gene numbers and high levels of duplication when using BRAKER and this could be caused by predicted isoforms of the same gene.

 Overall, I think the release of this well-assembled and annotated genome is a very useful contribution and I recommend the indexing of this article.

- Is the rationale for creating the dataset(s) clearly described?

Yes

- Are the protocols appropriate and is the work technically sound?

Yes

- Are sufficient details of methods and materials provided to allow replication by others?

Yes, but please include BUSCO scores for annotation

- Are the datasets clearly presented in a useable and accessible format?

Yes

Are sufficient details of methods and materials provided to allow replication by others?

Partly

Is the rationale for creating the dataset(s) clearly described?

Yes

Are the datasets clearly presented in a useable and accessible format?

Yes

Are the protocols appropriate and is the work technically sound?

Yes

Reviewer Expertise:

Evolutionary Biology

I confirm that I have read this submission and believe that I have an appropriate level of expertise to confirm that it is of an acceptable scientific standard.

# Peer review: Reviewer response for version 1

**DOI:** 10.21956/wellcomeopenres.21770.r63431

**Published:** 2023-07-31

**Authors:** Yue Yu

**Affiliations:** 1 Tufts University, Medford, Massachusetts, USA

The article is a clear and concise report on a genome assembly of the Light Brocade moth, *Lacanobia w-latinum*. The species is documented to have advanced spring phenology associated with climate change, and the authors point out that the genome sequence would be useful for studying the species' response to climate change as well as adaptation to calcareous habitats. Cutting-edge sequencing technologies are employed to assemble long scaffolds (using PacBio) with chromosomal/interaction information (using Hi-C). The resulting assembly appears to be of high quality (e.g. BUSCO 99%) and provides a solid reference genome for future studies.

 While the report is overall succinct, a few places would benefit from more details for reproducibility, completeness, and interpretation of data. For example:

1. Methods mentioned tissue set aside for Hi-C sequencing. What was the tissue (e.g. head)? Similarly, what was the tissue used for RNA extraction?
2. What parameters were used in the software tools? Even if all were at default values, explicitly stating this might be helpful for the readers.
3. How was the RNA-Seq data analysed? Additionally, it was unclear whether the RNA-Seq data was used for genome annotation. I could be wrong but as I understand it, BRAKER2 in the default protein mode is for when there is no transcriptomic data available.
4. While quality metrics for the final assembly were provided, I would be interested to see a summary of the sequencing and mapping quality. I could find some, but not all, of this information in the link provided.

Minor comment:

 Typo/missing word in Abstract: "chromosomal, "

Are sufficient details of methods and materials provided to allow replication by others?

Partly

Is the rationale for creating the dataset(s) clearly described?

Yes

Are the datasets clearly presented in a useable and accessible format?

Yes

Are the protocols appropriate and is the work technically sound?

Yes

Reviewer Expertise:

Population genomics, evolutionary genetics

I confirm that I have read this submission and believe that I have an appropriate level of expertise to confirm that it is of an acceptable scientific standard.
